# Transcript-Level Expression Patterns of Necroptosis-Related Genes RIPK1, RIPK3, and MLKL in Surgically Resected Non-Small Cell Lung Cancer: An Exploratory Single-Center Study

**DOI:** 10.3390/ijms27188361

**Published:** 2026-09-19

**Authors:** Fatma Ilknur Ulugun, Tuğba Yavuzşen, Safiye Aktaş, Zekiye Sultan Altun, Nezih Özdemir, Gökçen Ömeroğlu Şimşek, Duygu Gürel, Burçin Baran, Mehmet Emin Arayici

**Affiliations:** 1 Department of Thoracic Surgery, Faculty of Medicine, Dokuz Eylül University, 35330Izmir, Türkiye; nezih.ozdemir@deu.edu.tr; 2 Department of Medical Oncology, Oncology Institute, Dokuz Eylül University, 35330 Izmir, Türkiye; tugba.yavuzsen@deu.edu.tr; 3 Department of Basic Oncology, Oncology Institute, Dokuz Eylül University, 35330 Izmir, Türkiye; safiye.aktas@deu.edu.tr (S.A.); zekiyesaltun@gmail.com (Z.S.A.); burcinbrn@gmail.com (B.B.); 4 Department of Respiratory Disease, Faculty of Medicine, Dokuz Eylül University, 35330 Izmir, Türkiye; gokcen.simsek@deu.edu.tr; 5 Department of Pathology, Faculty of Medicine, Dokuz Eylül University, 35330 Izmir, Türkiye; gureld71@gmail.com; 6 Department of Biostatistics and Medical Informatics, Faculty of Medicine, Dokuz Eylül University, 35330 Izmir, Türkiye; mehmet.e.arayici@gmail.com

**Keywords:** non-small cell lung cancer, necroptosis, *RIPK1*, *RIPK3*, *MLKL*, prognosis

## Abstract

Necroptosis is a regulated inflammatory form of programmed cell death mediated by *RIPK1*, *RIPK3*, and *MLKL*, but its clinical relevance in surgically resected non-small cell lung cancer (NSCLC) remains uncertain. This study evaluated the transcript-level expression of these genes and their associations with survival in 41 patients who underwent complete NSCLC resection without preoperative therapy. RNA was isolated from frozen tumor tissues, and mRNA expression was quantified using RT-qPCR. Expression was analyzed primarily as continuous log_2_(expression + 1)-transformed variables in Cox proportional hazards models; median-based groups were used only for exploratory Kaplan–Meier visualization. The cohort comprised 34 males and 7 females, with a mean age of 61.0 years; 41.5% had adenocarcinoma and 58.5% had squamous cell carcinoma. The maximum follow-up was 145 months, and the 5-year overall survival rate was 46.3%. None of the three genes was significantly associated with overall or disease-free survival in either univariable or restricted adjusted Cox models. Median-based analyses similarly showed no significant survival differences. These findings do not support *RIPK1*, *RIPK3*, or *MLKL* transcript levels as standalone prognostic biomarkers in surgically resected NSCLC. Larger studies integrating transcriptomic, protein-level, and functional assessments are warranted.

## 1. Introduction

Lung cancer is the leading cause of cancer-related mortality worldwide and represents a major global health burden [1]. It is strongly linked to several established risk factors, particularly tobacco smoking, as well as passive smoke exposure, radon, asbestos, and other environmental or occupational carcinogens. The principal histological subtypes of non-small cell lung cancer (NSCLC) are adenocarcinoma (AC), squamous cell carcinoma (SCC), and large cell carcinoma, with AC and SCC representing the vast majority of cases [2]. Despite substantial advances in surgical techniques, perioperative management, systemic therapies, and multimodal treatment strategies, overall survival outcomes in lung cancer remain unsatisfactory. This persistent mortality is largely driven by pronounced biological heterogeneity and frequent diagnosis of the disease at advanced stages [3].

Clinical outcomes in NSCLC are strongly determined by the tumor stage and the feasibility of complete surgical resection. Five-year survival rates following curative resection range from nearly 75% in stage IA disease to approximately 25% in stage IIIA disease [4]. Nevertheless, nearly 70% of patients are diagnosed with locally advanced or metastatic disease, substantially limiting curative treatment options and contributing to persistently high mortality rates [5]. In particular, patients with stage IV metastatic NSCLC exhibit extremely poor outcomes, with approximately one-quarter to one-third dying within three months despite contemporary multimodal treatment regimens [6]. Against this backdrop of limited therapeutic success, understanding the molecular determinants of NSCLC progression has become increasingly imperative.

Among the emerging pathways of interest, necroptosis is a regulated, caspase-independent form of inflammatory cell death that functions as an alternative to apoptosis upon death receptor activation. This process is mediated by the receptor-interacting protein kinase 1 (RIPK1)–receptor-interacting protein kinase 3 (RIPK3)–mixed lineage kinase domain-like protein (MLKL) signaling cascade, which proceeds independently of caspase activation [7,8]. While necroptosis may serve as a compensatory mechanism in apoptosis-deficient cells, the dysregulation of necroptotic signaling has been implicated in malignant transformation and tumor progression [9].

Accumulating evidence indicates that necroptosis exerts context-dependent effects in cancer biology, influencing tumor growth, metastatic dissemination, and therapeutic responsiveness [10]. Necroptosis is associated with the release of diverse damage-associated molecular patterns (DAMPs), which in turn stimulate the production of inflammatory cytokines and chemokines, thereby modulating immune responses in multiple cancer types [11].

Consistent with this concept, necroptosis-related gene expression has been associated with prognosis in patients with NSCLC [12,13,14,15]. Previous studies have demonstrated that necroptosis-based gene expression signatures can predict overall survival and enable the stratification of patients into distinct molecular risk categories [13,14]. In NSCLC, disease progression and treatment response—particularly in the era of immunotherapy—are closely linked to immune surveillance, the tumor mutational burden, and immune checkpoint activity, with necroptosis-related gene signatures reflecting the dynamic interaction between regulated cell death pathways and the tumor immune microenvironment [12,15]. At the individual gene level, the reduced expression of key necroptotic regulators, including *RIPK1*, *RIPK3*, and *MLKL*, has been associated with early recurrence and unfavorable outcomes in NSCLC [16], whereas specific genetic variants in *RIPK1* and *MLKL* have been associated with improved survival following surgical resection [17].

Recent studies have further highlighted the potential clinical relevance of necroptosis-related gene expression in NSCLC [18]. Emerging evidence suggests that necroptosis-related pathways may contribute to tumor progression, invasion, and therapeutic responsiveness, supporting further investigation of core necroptosis regulators in lung cancer biology [19,20].

Although necroptosis-related gene signatures and transcriptomic associations have increasingly been reported in NSCLC, studies specifically focusing on the core necroptosis regulators *RIPK1*, *RIPK3*, and *MLKL* remain relatively limited, particularly in uniformly resected surgical cohorts. Therefore, in the present study, we quantified *RIPK1*, *RIPK3*, and *MLKL* mRNA expression levels using RT-qPCR in surgically resected NSCLC tissues and examined their transcript-level associations with disease-free and overall survival, using continuous expression analyses as the primary analytical approach.

## 2. Results

### 2.1. Characteristics of Patients

A total of 41 patients with NSCLC who underwent surgical resection were included in this study, comprising 34 males (82.9%) and 7 females (17.1%), and the mean age was 61.0 ± 7.65 years (median: 62 years; range: 48–75 years). Histologically, 17 patients (41.5%) had adenocarcinoma (AC), whereas 24 patients (58.5%) had squamous cell carcinoma (SCC). Regarding smoking status, 92.7% of patients were ever-smokers, whereas 7.3% were never-smokers. Pathologically, 34.1% of patients were Stage I, 34.1% Stage II, and 31.7% Stage IIIA. Patients were followed from the date of surgery until death or last follow-up. The median follow-up duration, estimated using the reverse Kaplan–Meier method, was 141.0 months (95% CI: 136.0–142.0 months), with a maximum follow-up duration of 145 months. During the follow-up period, 28 patients (68.3%) died, whereas 13 patients (31.7%) were alive at the last follow-up evaluation (Table 1). The postoperative systemic treatment consisted exclusively of platinum-based chemotherapy. In total, 27 patients received adjuvant chemotherapy, whereas 14 did not. According to the stage, chemotherapy was administered to 4 of 14 stage I patients, 13 of 14 stage II patients, and 10 of 13 stage III patients.

The mRNA expression levels of *RIPK1*, *RIPK3*, and *MLKL* in tumor tissues were quantified using RT-qPCR and were subsequently evaluated in the survival analyses described below.

### 2.2. Survival Analyses According to Continuous and Median-Based RIPK1, RIPK3, and MLKL Expression

The gene expression levels of *RIPK1*, *RIPK3*, and *MLKL* were analyzed primarily as continuous log_2_(expression + 1)-transformed variables. In these primary continuous-variable Cox analyses, none of the three genes showed statistically significant associations with OS or DFS in either univariable or restricted multivariable models (Supplementary Table S1). These findings indicated that the transcript-level expression of *RIPK1*, *RIPK3*, and *MLKL* did not demonstrate a robust continuous association with survival outcomes in the present cohort. These associations remained statistically non-significant after additional adjustment for adjuvant chemotherapy status.

**Figure 1:**
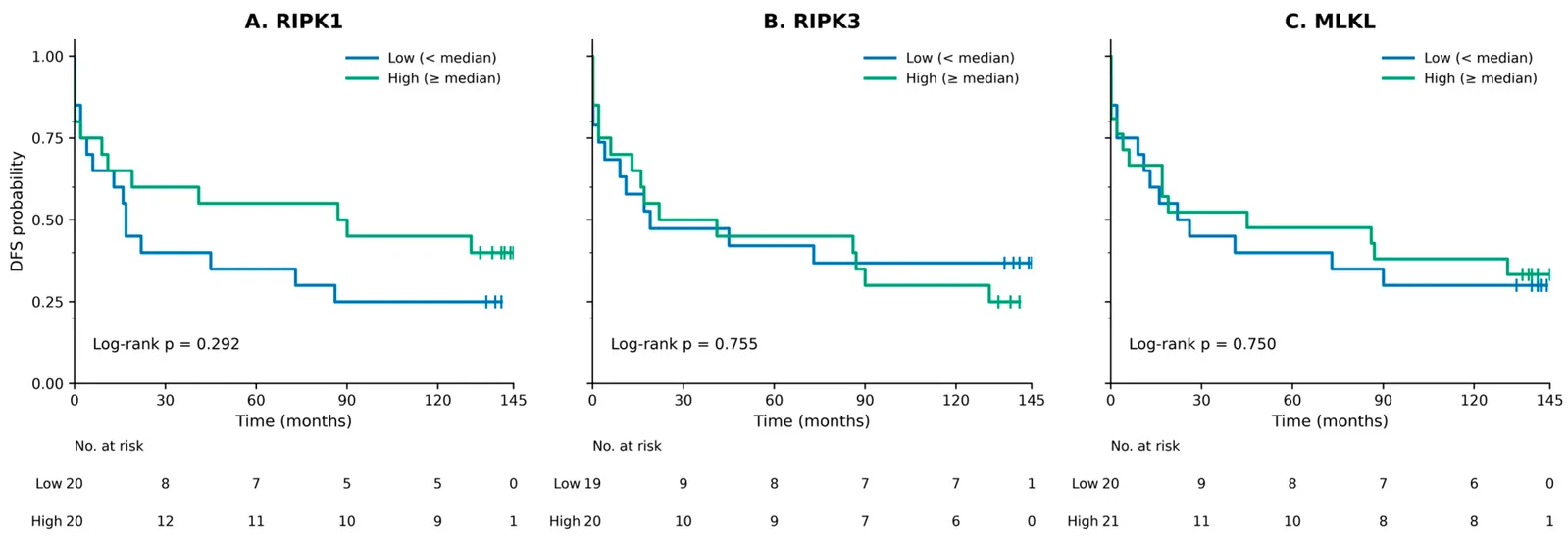
Disease-free survival according to median-based expression of *RIPK1*, *RIPK3*, and *MLKL* in the overall NSCLC cohort. Kaplan–Meier curves show DFS for (**A**) *RIPK1*, (**B**) *RIPK3*, and (**C**) *MLKL*. Patients were classified into low (<median) and high (≥median) expression groups using gene-specific median values (*RIPK1*: 0.3895; *RIPK3*: 0.2930; *MLKL*: 58.4710). Vertical tick marks indicate censored observations, and numbers at risk are presented below each panel. p values were calculated using the log-rank test.

**Figure 2:**
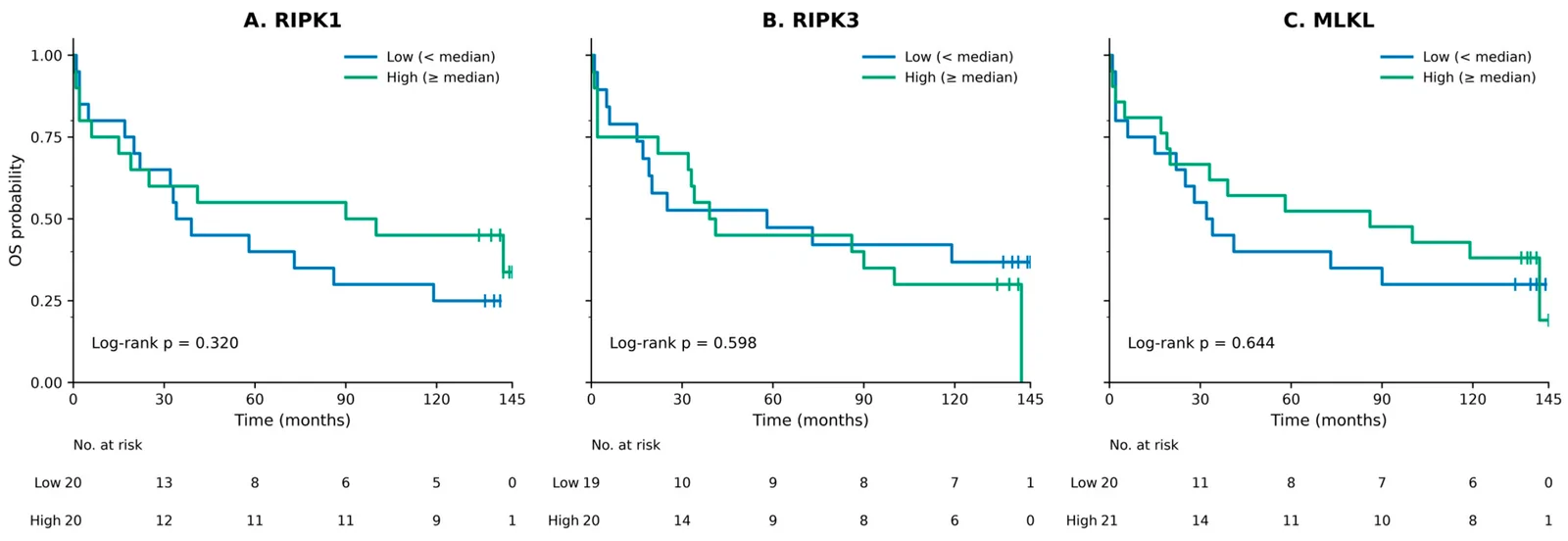
Overall survival according to median-based expression of *RIPK1*, *RIPK3*, and *MLKL* in the overall NSCLC cohort. Kaplan–Meier curves show OS for (**A**) *RIPK1*, (**B**) *RIPK3*, and (**C**) *MLKL*. Patients were classified into low (<median) and high (≥median) expression groups using gene-specific median values (*RIPK1*: 0.3895; *RIPK3*: 0.2930; *MLKL*: 58.4710). Vertical tick marks indicate censored observations, and numbers at risk are presented below each panel. p values were calculated using the log-rank test.

For exploratory Kaplan–Meier visualization, patients were additionally classified into low (<median) and high (≥median) expression groups. The median expression values were 0.3895 for *RIPK1*, 0.2930 for *RIPK3*, and 58.4710 for *MLKL*. No statistically significant differences in OS or DFS were observed for *RIPK1* (OS, *p* = 0.320; DFS, *p* = 0.292), *RIPK3* (OS, *p* = 0.598; DFS, *p* = 0.755), or *MLKL* (OS, *p* = 0.644; DFS, *p* = 0.750). These analyses were performed solely for exploratory visualization and were not intended to establish clinically applicable prognostic thresholds. The corresponding DFS and OS curves are presented in Figure 1 and Figure 2, respectively.

### 2.3. Exploratory Analyses of Publicly Available Datasets

DepMap CRISPR/Chronos dependency scores for *RIPK1*, *RIPK3*, and *MLKL* were centered near zero across NSCLC cell lines, including LUAD (lung adenocarcinoma)- and LUSC (lung squamous cell carcinoma)-like lineages. These findings did not indicate a strong lineage-specific dependency on any of the three genes (Supplementary Table S2). TCGA-based UALCAN analyses showed no significant differences in *RIPK1*, *RIPK3*, or *MLKL* expression between primary tumors and normal lung tissues in LUAD (*p* = 0.065, *p* = 0.636, and *p* = 0.425, respectively). In LUSC, the expression levels of all three genes were significantly lower in primary tumors than in normal lung tissues (*RIPK1*, *p* = 1.56 × 10^−10^; *RIPK3*, *p* < 1 × 10^−12^; *MLKL*, *p* = 1.62 × 10^−12^). *RIPK1* expression was not significantly associated with survival in either LUAD or LUSC (*p* = 0.19 and *p* = 0.13, respectively). Higher *RIPK3* expression was associated with poorer survival in both LUAD (*p* = 0.029) and LUSC (*p* = 0.005), whereas higher *MLKL* expression was associated with poorer survival in LUAD (*p* = 0.0032) and showed a non-significant adverse trend in LUSC (*p* = 0.068). These publicly available dataset analyses were considered contextual and were not interpreted as direct external validation of the present cohort (Supplementary Table S3).

## 3. Discussion

In this exploratory single-center study of patients with surgically resected NSCLC, the principal finding was that the transcript-level expression of *RIPK1*, *RIPK3*, and *MLKL* was not significantly associated with OS or DFS when analyzed as continuous log_2_(expression + 1)-transformed variables in either univariable or restricted adjusted Cox models. Consistent with these findings, the exploratory median-based Kaplan–Meier analyses showed no statistically significant survival differences for any of the three genes. Therefore, the present results do not support the use of *RIPK1*, *RIPK3*, or *MLKL* mRNA expression as standalone prognostic biomarkers in this cohort. Given the limited sample size and single-center design, however, the absence of statistically significant associations should not be interpreted as evidence that these genes lack biological relevance in NSCLC. Rather, the findings indicate that any transcript-level prognostic effects, if present, could not be demonstrated robustly in the present cohort and require evaluation in larger, independent patient populations.

The present findings differ from those reported by Park et al., who observed that reduced expression of key necroptosis-related genes was associated with poorer DFS in patients with AC, whereas no significant prognostic association was identified in SCC [16]. In contrast, neither the primary continuous-variable analyses nor the exploratory median-based analyses in the present cohort demonstrated significant associations between *RIPK1*, *RIPK3*, or *MLKL* expression and survival outcomes. This discrepancy may reflect differences in cohort size and composition, tissue selection, gene-expression quantification, follow-up characteristics, and statistical methodology. Moreover, the limited sample size of the present cohort did not permit sufficiently reliable histology-specific survival estimates. Collectively, these differences highlight the context-dependent nature of necroptosis-related gene expression and the need for validation in larger, histologically well-characterized NSCLC cohorts.

The analyses of publicly available datasets provided a broader functional and transcriptomic context but did not constitute an external validation of the present cohort. DepMap CRISPR/Chronos dependency scores for *RIPK1*, *RIPK3*, and *MLKL* were generally centered near zero in NSCLC cell lines, with no evidence of a strong lineage-specific dependency in either LUAD- or LUSC-like models. In the TCGA/UALCAN analyses, none of the three genes showed a significant tumor-versus-normal expression difference in LUAD, whereas the expression levels of all three genes were significantly lower in LUSC tumors than in normal lung tissue. Survival patterns were also context-dependent. Higher *RIPK3* and *MLKL* expression was associated with poorer survival in LUAD, whereas *RIPK1* was not significantly associated with survival. In LUSC, higher RIPK3 expression was associated with poorer survival, *MLKL* showed a non-significant adverse trend, and *RIPK1* again showed no significant survival association. These findings were not concordant with a consistent prognostic direction across histological subtypes or datasets. This variability suggests that transcript abundance alone may not directly represent functional necroptotic activity and that observed survival associations may be influenced by tumor histology, cellular composition, clinical characteristics, treatment exposure, and analytical platform. Accordingly, the public database results should be regarded as contextual evidence rather than independent confirmation of the present findings (Supplementary Tables S2 and S3).

Beyond NSCLC, the prognostic relevance of *MLKL* has been investigated in several malignancies. *MLKL* expression has been associated with clinical outcomes in gastric and pancreatic cancers [21,22], while a meta-analysis identified reduced *MLKL* expression as an adverse prognostic factor across multiple cancer types [23]. Although these studies support the broader biological and prognostic relevance of *MLKL*, their findings cannot be directly extrapolated to surgically resected NSCLC. In the present cohort, *MLKL* expression was not significantly associated with OS or DFS in either the primary continuous-variable analyses or the exploratory median-based analyses. Differences between the present findings and the previous literature may reflect tumor-specific biology, cohort characteristics, treatment patterns, analytical methodology, and the distinction between transcript-level expression and functional protein activity. Thus, the available evidence provides a rationale for further investigation of *MLKL* but does not establish its transcript-level expression as a prognostic biomarker in resected NSCLC.

Previous studies in NSCLC have reported associations between necroptosis-related regulators and clinical outcomes and have suggested potential interactions between necroptotic signaling and the tumor immune microenvironment [16,24]. In the present cohort, however, transcript-level expression of *RIPK1*, *RIPK3*, and *MLKL* was not significantly associated with survival outcomes. This discrepancy underscores that mRNA abundance alone may not reliably indicate functional activation of the necroptosis pathway. Functional necroptotic signaling depends not only on the expression of its core regulators but also on post-translational activation, protein complex formation, and the phosphorylation and oligomerization of MLKL. Because these processes were not evaluated, the present transcript-level findings neither confirm nor exclude a biological role for necroptosis in resected NSCLC. Future studies integrating gene expression with protein-level, phosphorylation, immune-profile, and functional assessments are therefore needed.

At the genetic level, previous studies have also reported histology-dependent associations involving variants of necroptosis-related genes. For example, the MLKL rs877375G>C variant has been associated with increased promoter activity, higher mRNA expression, and more favorable clinical outcomes in patients with AC, whereas a comparable association was not demonstrated in SCC [17]. These observations suggest that the regulation and clinical relevance of necroptosis-related pathways may vary according to tumor context and histological subtype. However, genotyping was not performed in the present study, and the limited cohort size precluded reliable histology-specific inference. Therefore, the present transcript-level findings cannot be directly linked to previously reported genetic associations.

Necroptosis is increasingly recognized as a context-dependent process with both tumor-suppressive and tumor-promoting effects [25]. When apoptotic signaling is impaired, necroptosis may function as an alternative regulated cell-death pathway or as a compensatory “fail-safe” mechanism. Conversely, membrane disruption and the release of damage-associated molecular patterns may generate inflammatory responses that either enhance antitumor immunity or support tumor progression, depending on the cellular and immune context [11,25]. This biological duality may partly explain why associations between necroptosis-related gene expression and survival vary across tumor types, histological subtypes, and patient cohorts, as also suggested by the non-concordant patterns observed across the present cohort and the publicly available datasets. Because the current study assessed mRNA expression alone, it was not possible to determine whether the necroptosis pathway was functionally active or to characterize its downstream inflammatory consequences. Future translational studies integrating transcriptomic data with total and phosphorylated protein expression, spatial immune profiling, and functional pathway assessments are needed to clarify the clinical relevance of necroptosis in resected NSCLC [26,27,28,29,30,31].

This study has several limitations. The sample size was relatively small, and the retrospective single-center design may have introduced selection bias and limited the generalizability of the findings. Detailed information on smoking intensity, smoking duration, comorbidities, and treatment regimens was not uniformly available, which may have limited adjustment for residual confounding.

Gene expression was evaluated only at the mRNA level using RT-qPCR. Protein-level analyses, such as immunohistochemistry, Western blotting, and phosphorylated MLKL (p-MLKL) assessments, were not performed and could provide important complementary information. Similarly, the present study did not include analyses of immune cell infiltration, inflammatory cytokines, or immune checkpoint expression. Therefore, interpretations regarding the tumor immune microenvironment remain speculative.

Adjacent non-malignant lung tissue was not available as a biological control. As a result, we could not determine whether *RIPK1*, *RIPK3*, and *MLKL* were specifically upregulated or downregulated relative to normal lung tissue. The findings should therefore be interpreted as transcript-level associations within tumor tissue rather than tumor-versus-normal comparisons.

Finally, the limited number of patients and outcome events constrained the complexity and precision of the Cox regression models. Restricted adjustment was therefore used to reduce the risk of overfitting, although residual confounding by unmeasured or incompletely recorded clinical factors cannot be excluded. Missing expression measurements resulted in gene-specific complete-case sample sizes, further reducing statistical power. The median-based Kaplan–Meier analyses were performed only for exploratory visualization and should not be interpreted as establishing clinically applicable prognostic thresholds. Moreover, the publicly available dataset analyses provided contextual evidence rather than direct external validation. Larger, multicenter cohorts with independent validation and integrated transcriptomic, protein-level, and functional assessments are required to determine whether *RIPK1*, *RIPK3*, or *MLKL* has prognostic relevance in resected NSCLC.

## 4. Materials and Methods

### 4.1. Patient Population

A total of 41 patients diagnosed with NSCLC who underwent surgery between January 2013 and September 2013 were enrolled in this study. During the study period, patients were included according to the availability of frozen tumor tissue suitable for RNA extraction. The final cohort therefore represents a tissue-availability-based retrospective cohort rather than a population-based surgical series. Cancers were staged according to the 8th edition Tumor–Node–Metastasis staging system [32]. None of the patients had undergone chemotherapy or radiotherapy prior to surgery. Only patients who underwent complete tumor resection (R0 resection) were included. Patients with inflammatory diseases who had undergone limited resection (wedge resection or segmentectomy) and had a history of other malignancies were excluded. Overall survival (OS) was defined as the time from surgery to death from any cause or last follow-up. Disease-free survival (DFS) was defined as the time from surgery to recurrence or death, whichever occurred first, or last follow-up. As this cohort was treated in 2013, postoperative systemic treatment was limited to platinum-based chemotherapy; notably none of the patients received targeted therapy. Adjuvant chemotherapy was administered from stage IB onward according to multidisciplinary tumor board decisions and the prevailing clinical practice of that period. All frozen fresh tumors were collected in accordance with protocols approved by the institutional review board. Tumor-containing tissue areas were selected macroscopically by the pathologist prior to analysis. Written informed consent was obtained at the time of sample collection in 2013. In addition, consent specific to the present retrospective study was obtained from surviving patients or from the relatives/legal representatives of deceased patients. This study was approved by the university’s Ethics Committee (2024/36-01).

### 4.2. Gene Expression Analysis

Total RNA was isolated from frozen tumor tissue samples using the High Pure RNA Isolation Kit (Roche Diagnostics GmbH,, cat. no. 11828665001, Mannheim, Germany). RNA concentrations were measured using a Qubit fluorometer (Thermo Fisher Scientific, Waltham, MA, USA). Next, RNA was converted to cDNA using a reverse transcriptase kit (Applied Biological Materials Inc. [abm], Richmond, BC, Canada). The quantitative real-time PCR (RT-qPCR) reaction was carried out with SYBR Green Master Mix (Roche LightCycler 480, Roche Diagnostics GmbH, Mannheim, Germany) using specific primers (*RIPK1* forward, 5′ AGC CGA GAT GAG TAC TCC G 3′; *RIPK1* reverse, 5′ TCC TTG TGT ATC ACG CCT TT 3′; RIPK3 forward, 5′ CCT TCC GGT TTA CGT TAA CAA AC 3′; *RIPK3* reverse, 5′ ACG TCA AGC TGG CAG ATT TTG 3′; *MLKL* forward 5′ CCA GAT GCT AAG AAG AGA TAA TGA A 3′; *MLKL* reverse, 5′AAA TAC TGC CTC AAA GTT TCC T 3′; *B-actin* (*ACTB*) (control primer) forward 5′ TGA GCG CGG CTA CAG CTT 3′; reverse 5′ TCC TTA ATG TCA CGC ACG CAC GAT TT 3′) in a Nano (Roche) machine. RT-qPCR reactions were performed in a total volume of 20 μL containing 10 μL SYBR Green Master Mix (Thermo Fisher Scientific), 2 μL primer mixture (10 μM each), 5 μL cDNA template, and 3 μL nuclease-free water. Amplification was carried out using a Roche LightCycler 96 platform under the following cycling conditions: initial denaturation at 95 °C for 5 s, followed by 45 cycles of denaturation at 95 °C for 10 s, annealing at 55 °C for 20 s, and elongation at 72 °C for 20 s. Relative gene expression levels were calculated using the Livak and Schmittgen (2^−ΔΔCt^) method [33].

### 4.3. Statistical Analysis

All statistical analyses were performed using R version 4.5.3 (R Foundation for Statistical Computing, Vienna, Austria), IBM SPSS Statistics, version 30.0 (IBM Corp., Armonk, NY, USA), and JAMOVI, version 2.7.4 (The JAMOVI Project, Sydney, Australia). Continuous variables were summarized using means with standard deviations or medians with interquartile ranges, as appropriate, whereas categorical variables were presented as frequencies and percentages. The expression levels of RIPK1, RIPK3, and MLKL were analyzed primarily as continuous variables after log_2_(expression + 1) transformation. Each gene was evaluated separately in univariable Cox proportional hazards models for disease-free survival (DFS) and overall survival (OS). Given the limited cohort size and number of events, restricted adjusted models were used to avoid overfitting. Model 1 included the transformed gene expression value, pathological stage (stage II–III versus stage I), and histological subtype (squamous cell carcinoma versus adenocarcinoma). Model 2 additionally included adjuvant chemotherapy status (no adjuvant chemotherapy versus receipt of adjuvant chemotherapy). Hazard ratios (HRs) for gene expression were reported per one-unit increase in the log_2_(expression + 1)-transformed value, together with their corresponding 95% confidence intervals (CIs). Analyses were conducted using complete cases available for each gene, and missing expression values were not imputed.

For exploratory Kaplan–Meier visualization, patients were additionally classified into low (<median) and high (≥median) expression groups using the gene-specific median values: 0.3895 for *RIPK1*, 0.2930 for *RIPK3*, and 58.4710 for *MLKL*. Survival curves were compared using the log-rank test. These median-based groupings were used solely for graphical exploration and were not considered primary prognostic analyses or clinically validated expression thresholds. Median follow-up duration was estimated using the reverse Kaplan–Meier method. All statistical tests were two-sided. A nominal *p* value < 0.05 was considered statistically significant; however, given the exploratory nature and limited sample size of the study, the findings were interpreted cautiously.

## 5. Conclusions

In this exploratory single-center cohort, the transcript-level expression of *RIPK1*, *RIPK3*, and *MLKL* was not significantly associated with DFS or OS when evaluated as continuous variables or using median-based exploratory Kaplan–Meier analyses. Accordingly, the present findings do not support the use of these genes as standalone prognostic biomarkers in surgically resected NSCLC. Nevertheless, the biological and clinical relevance of necroptosis cannot be excluded, because mRNA abundance may not reflect protein activation or pathway functionality, and the statistical power of this study was limited. Larger multicenter studies integrating transcriptomic, protein-level, immune microenvironment, and functional assessments are needed to clarify the context-dependent prognostic role of necroptosis in NSCLC.

## Figures and Tables

**Table 1:** The clinicopathological characteristics of the patients (n = 41).

Variable	Value
**Age, years, mean ± SD (range)**	61 ± 7.65 (48–75)
**Male sex, n (%)**	34 (82.9%)
**Histology, n (%)**	
AC	17 (41.5%)
SCC	24 (58.5%)
**Stage, n (%)**	
I	14 (34.1%)
II	14 (34.1%)
IIIA	13 (31.7%)
**Survival status, n (%)**	
Dead	28 (68.3%)
Alive	13 (31.7%)
**Smoking status, n (%)**	
Ever-smoker	38 (92.7%)
Never-smoker	3 (7.3%)
**Surgery type, n (%)**	
Lobectomy	33 (80.5%)
Pneumonectomy	8 (19.5%)
**Lymphovascular invasion, n (%)**	
Yes	22 (53.7%)
No	19 (46.3%)
**Perineural invasion, n (%)**	
Yes	17 (41.5%)
No	24 (58.5%)
**Visceral pleural invasion, n (%)**	
Yes	12 (29.3%)
No	29 (70.7%)

## Data Availability

The data that support the findings of this study are available from the corresponding author upon reasonable request.

## Supplementary Materials

The following supporting information can be downloaded at: https://www.mdpi.com/article/10.3390/ijms27188361/s1.

## Abbreviations

The following abbreviations are used in this manuscript:

AC	Adenocarcinoma
ACTB	Beta-actin
CI	Confidence interval
DAMPs	Damage-associated molecular patterns
DFS	Disease-free survival
HR	Hazard ratio
IQR	Interquartile range
MLKL	Mixed lineage kinase domain-like protein
mRNA	Messenger RNA
NSCLC	Non-small cell lung cancer
OS	Overall survival
p-MLKL	Phosphorylated mixed lineage kinase domain-like protein
RT-qPCR	Reverse transcription quantitative polymerase chain reaction
SCC	Squamous cell carcinoma
SD	Standard deviation
TCGA	The Cancer Genome Atlas
TNM	Tumor–Node–Metastasis
UALCAN	University of Alabama at Birmingham Cancer Data Analysis Portal
