# Supplementary material for: Transcript-Level Expression Patterns of Necroptosis-Related Genes RIPK1, RIPK3, and MLKL in Surgically Resected Non-Small Cell Lung Cancer: An Exploratory Single-Center Study
Source: Int J Mol Sci. 2026 Sep 19;27(18):8361. doi: 10.3390/ijms27188361 (PMC13607173; doi:10.3390/ijms27188361)
Supplement: Supplementary file 1 [file ijms-27-08361-s001.zip › ijms-4551899-supplementary.pdf]

Supplementary Tables

Table S1. Primary Cox regression analyses evaluating RIPK1, RIPK3, and MLKL mRNA expression as continuous $\log_2$(expression + 1)-transformed variables.

Endpoint	Gene	Model	n	Events	HR	95% CI	p value
OS	*RIPK1*	Univariable	40	27	0.88	0.69–1.13	0.314
OS	*RIPK1*	Model 1	40	27	0.88	0.68–1.13	0.305
OS	*RIPK1*	Model 2	40	27	0.87	0.68–1.11	0.264
OS	*RIPK3*	Univariable	39	27	0.92	0.70–1.20	0.541
OS	*RIPK3*	Model 1	39	27	0.91	0.69–1.19	0.498
OS	*RIPK3*	Model 2	39	27	0.90	0.69–1.18	0.438
OS	*MLKL*	Univariable	41	28	0.94	0.84–1.05	0.296
OS	*MLKL*	Model 1	41	28	0.94	0.85–1.05	0.303
OS	*MLKL*	Model 2	41	28	0.94	0.84–1.05	0.280
DFS	*RIPK1*	Univariable	40	27	0.87	0.68–1.12	0.282
DFS	*RIPK1*	Model 1	40	27	0.87	0.68–1.12	0.275
DFS	*RIPK1*	Model 2	40	27	0.85	0.67–1.09	0.212
DFS	*RIPK3*	Univariable	39	27	0.89	0.67–1.17	0.396
DFS	*RIPK3*	Model 1	39	27	0.88	0.67–1.16	0.354
DFS	*RIPK3*	Model 2	39	27	0.85	0.64–1.12	0.243
DFS	*MLKL*	Univariable	41	28	0.96	0.86–1.07	0.431
DFS	*MLKL*	Model 1	41	28	0.96	0.86–1.07	0.441
DFS	*MLKL*	Model 2	41	28	0.96	0.86–1.07	0.428

Note: The reported HRs correspond to gene expression and are presented per one-unit increase in $\log_2$(expression + 1)-transformed mRNA expression. Model 1 was adjusted for pathological stage and histological subtype. Model 2 was additionally adjusted for adjuvant chemotherapy status. Pathological stage was coded as stage II–III versus stage I, histological subtype as squamous cell carcinoma versus adenocarcinoma, and adjuvant chemotherapy status as no adjuvant chemotherapy versus receipt of adjuvant chemotherapy. Analyses were conducted using complete cases available for each gene. DFS, disease-free survival; OS, overall survival; HR, hazard ratio; CI, confidence interval.

Table S2. DepMap-based external functional context of RIPK1, RIPK3, and MLKL in non-small cell lung cancer cell lines.

Gene	DepMap context	Observed dependency pattern	Brief interpretation
RIPK1	NSCLC	Dependency scores were centered near zero rather than in the strongly negative range	No strong NSCLC-specific dependency observed
RIPK3	NSCLC	Dependency scores remained close to zero without a marked shift toward strong negative dependency	No strong NSCLC-specific dependency observed
MLKL	NSCLC	Dependency scores were distributed near zero and did not indicate strong negative dependency	No strong NSCLC-specific dependency observed
RIPK1	LUAD-like cell lines	Scores remained centered near zero	No strong LUAD-specific dependency observed
RIPK1	LUSC-like cell lines	Scores remained centered near zero	No strong LUSC-specific dependency observed
RIPK3	LUAD-like cell lines	Scores remained centered near zero	No strong LUAD-specific dependency observed
RIPK3	LUSC-like cell lines	Scores remained centered near zero	No strong LUSC-specific dependency observed
MLKL	LUAD-like cell lines	Scores remained centered near zero	No strong LUAD-specific dependency observed
MLKL	LUSC-like cell lines	Scores remained centered near zero	No strong LUSC-specific dependency observed

Abbreviations: LUAD, lung adenocarcinoma; LUSC, lung squamous cell carcinoma; NSCLC, non-small cell lung cancer. DepMap interpretation was based on CRISPR/Chronos lineage- and context-specific dependency distributions.

Table S3. TCGA/UALCAN-based external transcriptomic and survival context of RIPK1, RIPK3, and MLKL in LUAD and LUSC.

Gene	TCGA cohort	Expression pattern	Survival association	Brief interpretation
RIPK1	LUAD	No significant difference between normal and primary tumor ($p = 0.065$)	No significant survival association ($p = 0.19$)	No clear tumor-normal or survival difference in LUAD
RIPK1	LUSC	Significantly lower in primary tumor than in normal tissue ($p = 1.56 \times 10^{-10}$)	No significant survival association ($p = 0.13$)	Downregulated in LUSC without significant survival separation
RIPK3	LUAD	No significant difference between normal and primary tumor ($p = 0.636$)	High expression associated with worse survival ($p = 0.029$)	No tumor-normal difference, but adverse survival association in LUAD
RIPK3	LUSC	Significantly lower in primary tumor than in normal tissue ($p < 1 \times 10^{-12}$)	High expression associated with worse survival ($p = 0.005$)	Downregulated in tumors, but higher expression linked to poorer survival in LUSC
MLKL	LUAD	No significant difference between normal and primary tumor ($p = 0.425$)	High expression associated with worse survival ($p = 0.0032$)	No tumor-normal difference, but adverse survival association in LUAD
MLKL	LUSC	Significantly lower in primary tumor than in normal tissue ($p = 1.62 \times 10^{-12}$)	Borderline survival association; high expression tended to worse survival ($p = 0.068$)	Downregulated in tumors, with a non-significant adverse trend

Abbreviations: LUAD, lung adenocarcinoma; LUSC, lung squamous cell carcinoma. Expression and survival patterns were explored using TCGA-based UALCAN analyses.
